# Atomic Force Microscopy on Biological Materials Related to Pathological Conditions

**DOI:** 10.1155/2019/8452851

**Published:** 2019-05-12

**Authors:** Andreas Stylianou, Stylianos-Vasileios Kontomaris, Colin Grant, Eleni Alexandratou

**Affiliations:** ^1^Cancer Biophysics Laboratory, Department of Mechanical and Manufacturing Engineering, University of Cyprus, Nicosia 2238, Cyprus; ^2^Mobile Radio Communications Laboratory, School of Electrical and Computer Engineering, National Technical University of Athens, Iroon Polytechniou, Athens 15780, Greece; ^3^Athens Metropolitan College, Sorou 74, Marousi 15125, Greece; ^4^Hitachi High-Technologies Europe, Techspace One, Keckwick Lane, Warrington WA4 4AB, UK; ^5^Biomedical Optics and Applied Biophysics Laboratory, School of Electrical and Computer Engineering, National Technical University of Athens, Iroon Polytechniou, Athens 15780, Greece

## Abstract

Atomic force microscopy (AFM) is an easy-to-use, powerful, high-resolution microscope that allows the user to image any surface and under any aqueous condition. AFM has been used in the investigation of the structural and mechanical properties of a wide range of biological matters including biomolecules, biomaterials, cells, and tissues. It provides the capacity to acquire high-resolution images of biosamples at the nanoscale and allows at readily carrying out mechanical characterization. The capacity of AFM to image and interact with surfaces, under physiologically relevant conditions, is of great importance for realistic and accurate medical and pharmaceutical applications. The aim of this paper is to review recent trends of the use of AFM on biological materials related to health and sickness. First, we present AFM components and its different imaging modes and we continue with combined imaging and coupled AFM systems. Then, we discuss the use of AFM to nanocharacterize collagen, the major fibrous protein of the human body, which has been correlated with many pathological conditions. In the next section, AFM nanolevel surface characterization as a tool to detect possible pathological conditions such as osteoarthritis and cancer is presented. Finally, we demonstrate the use of AFM for studying other pathological conditions, such as Alzheimer's disease and human immunodeficiency virus (HIV), through the investigation of amyloid fibrils and viruses, respectively. Consequently, AFM stands out as the ideal research instrument for exploring the detection of pathological conditions even at very early stages, making it very attractive in the area of bio- and nanomedicine.

## 1. Introduction

Atomic force microscopy (AFM) belongs to the scanning probe microscopy (SPM) family and was developed following on from the scanning tunnelling microscopy (STM), which was awarded the 1986 Nobel Prize in Physics. AFM is a SPM that records interactions between a sharp probe (the AFM tip) at the end of a small cantilever and the sample surface. Since its invention in the 1980s, it has become a fundamental technique in the fields of surface science. AFM has several advantages over the other microscopic techniques, such as scanning and transmission electron microscopy (SEM and TEM) and optical microscopy (including fluorescent and confocal laser scanning microscopy). First of all, AFM provides quantifiable and accurate surface height information, down to the Angstrom level—while other microscopes can give topographical contrast, they cannot provide three-dimensional topographies. Measurements and images captured by AFM can be made in air, aqueous, or vacuum conditions at a range of temperatures. Plus, the sample preparations are considerably easier than those used for TEM. After image acquisition, the AFM user can perform mechanical/electrical/magnetic property characterization of sample surface, offering a combination of qualitative and quantitative information [1]. AFM is characterized as a nondestructive tool that can operate under different conditions (air and liquid) since it requires only the basic sample preparation (e.g., does not require dehydration, labeling with fluorescent dyes or antibodies, or surface coating) [2–5].

**Figure 1:**
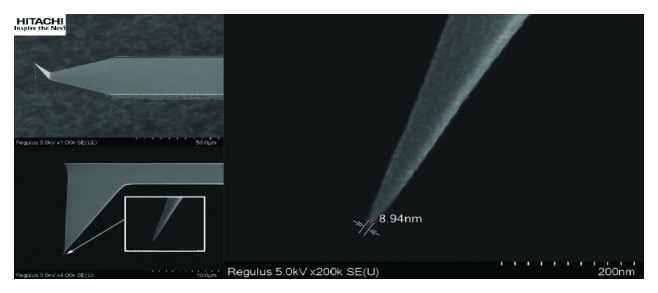
AFM tip. SEM images (Hitachi Regulus SU 8230) of an Olympus AC160 AFM probe, with a measured tip diameter of 9 nm (unpublished data obtained by Colin Grant).

AFM was developed in 1986 by Binnig and colleagues [6] and commercial AFMs began to appear in the early 1990s [7]. Since its invention, it has rapidly become a popular method for high-resolution nanoscale imaging and mechanical property characterization of a broad range of samples, especially biological materials [3, 8]. The key requirement for AFM imaging is the probe, a sharp tip mounted on a cantilever (Figure 1). A huge range of tip shapes and geometries are commercially available along with a range of cantilever spring constants (0.001 to 2000 N/m) and cantilever coatings that can allow imaging of delicate soft matter without causing damage or even make indentations in glass.

### 1.1. Force versus Distance Curves

**Figure 2:**
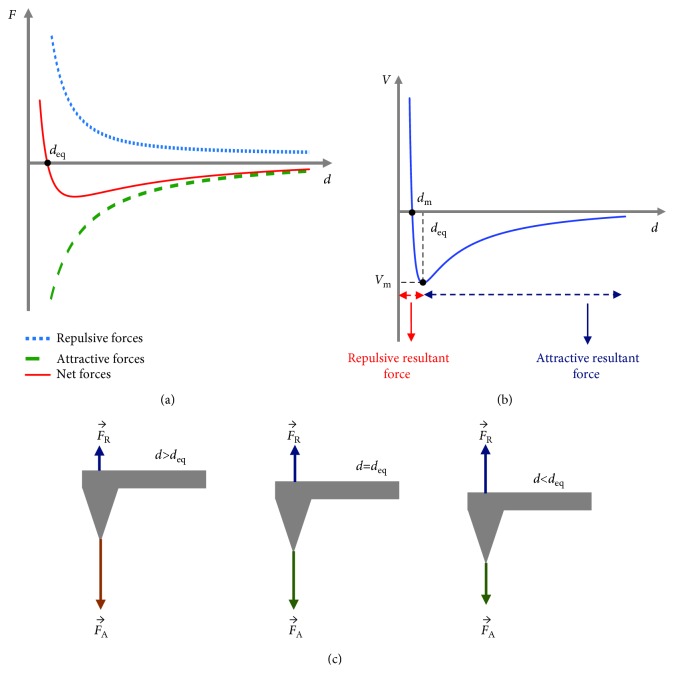
(a) Force-distance curves. Interaction forces versus distance between the tip and the sample's surface. (b) The Lennard–Jones potential. If *d* > *d*_eq_, the resultant force is attractive, and if *d* < *d*_eq_, the resultant force is repulsive. In the case that *d* = *d*_eq_, the resultant force is zero. (c) Repulsive and attractive forces on the tip. Forces applied on the AFM tip in three different regions.

In this section, the applied forces during the interaction between the AFM tip and the sample's surface will be presented. These forces are attractive or repulsive depending on the distance between the AFM tip and the sample (Figure 2(a)). More specifically, if the abovementioned distance is big enough, the resultant force is attractive (van der Waals force) [9]. On the contrary, for small distances, the resultant force is repulsive due to the overlapping of electron orbitals between the tip and sample [9]. The aforementioned forces can be approximated using the Lennard–Jones potential (Figure 2(b)) [10]:

(1) $$\begin{array}{c} V\left(d\right)=4V_{\text{m}}\left[{\left(\frac{d_{\text{m}}}{d}\right)}^{12}-{\left(\frac{d_{\text{m}}}{d}\right)}^{6}\right], \end{array}$$

where *V*(*d*) and *d* are the intermolecular potential and the distance between the two atoms or molecules, respectively; *V*_m_ is the well depth (a measure of how much the two particles attract each other); and *d*_m_ is the distance at which *V* = 0.

As it is presented in Figure 2(a), when the distance between the AFM tip and the sample's surface is equal to *d*_eq_ (where *d*_eq_ is the equilibrium distance), the resultant force on the tip is zero. In addition, in the case that *d* > *d*_eq_, the resultant force is attractive (negative net force, *F*_T_ = *F*_R_ − *F*_A_ < 0, where *F*_R_ is the repulsive force, *F*_A_ the attractive force, and *F*_T_ the total force on the tip). On the contrary, in the case that *d* < *d*_eq_, the resultant force is repulsive (positive net force, *F*_T_ = *F*_R_ − *F*_A_ > 0).

### 1.2. Applied Forces in the AFM Operation Modes

**Figure 3:**
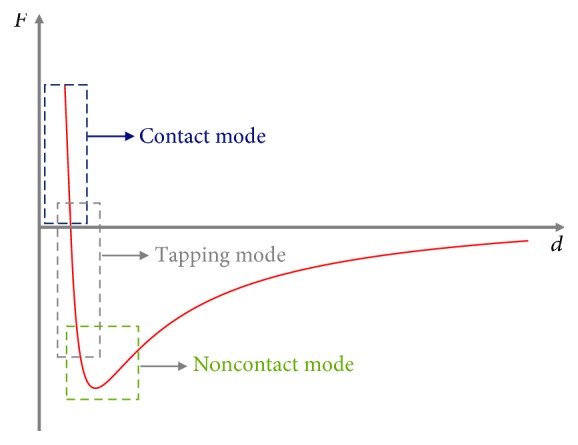
Force versus distance: the blue box represents the range of interaction forces in the contact mode (repulsive net force). The grey box and the green box represent the range of the interaction forces in the intermittent and in the noncontact regions, respectively.

The categorization of the imaging modes is based on the type of the applied forces on the sample. More specifically, in the contact mode, the tip is always in contact with the sample and the resultant force is repulsive. On the contrary, in the noncontact mode, the tip is never in contact with the sample's surface; thus, the resultant force is attractive. In the tapping mode, the resultant force can be attractive or repulsive since the tip alternately moves toward to or away from the sample's surface. Last but not least, in the force mode (in which the sample's mechanical properties must be tested), the sample is moving toward the tip; hence, the net force is initially attractive and then repulsive. The selection of an AFM imaging mode depends on the characteristics of the sample that must be tested. For example, for a nonbiological sample, the contact mode is preferable due to the fact that there is no possibility to cause permanent damage to the sample. In the case of very soft biological samples, the noncontact mode seems to be the preferred choice. However, it presents severe limitations (e.g., it must be applied in a high vacuum environment) [11, 12], and as a result, the tapping mode is mostly used for imaging soft biological samples. In Figure 3, the total force with respect to distance (between the tip and the sample) is presented. The blue box presents the range of interaction forces in the contact region, and the green and grey boxes the range of interaction forces in the noncontact region and the intermittent region (tapping mode), respectively.

**Figure 4:**
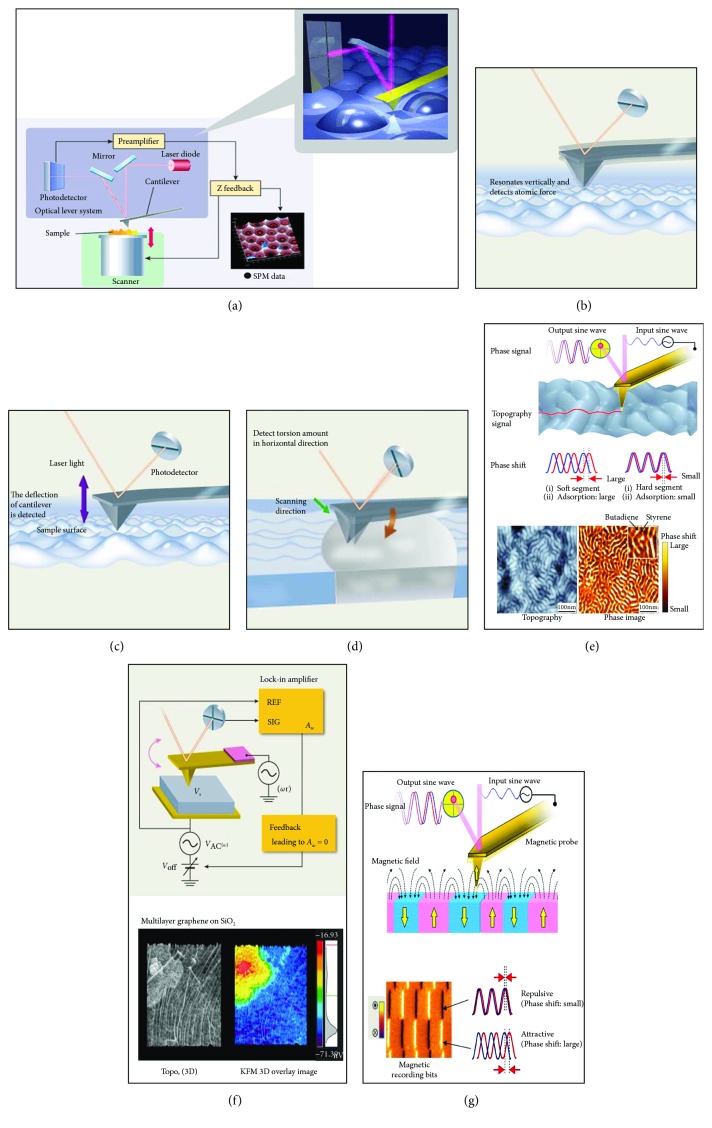
AFM working principles and modes: (a) generalized schematic of AFM of cantilever with the laser reflecting onto the photodetector. (b) Tapping/dynamic mode. (c) Contact mode. (d) Friction/lateral mode. (e) Phase imaging from dynamic mode, where the contrast (butadiene/styrene blend) relates to surface properties. (f) Kelvin probe electrical imaging; contrast shows surface potential (work function) of a graphene flake on SiO_2_ substrate. (g) Magnetic mode imaging, where a magnetic coated cantilever scans just above the surface and the phase shift relates to attraction/repulsion of magnetic domains (images courtesy of Hitachi High Technology, Tokyo, Japan).

The AFM instrument itself consists of a laser beam aligned to the back of a cantilever, which is then reflected onto a position-sensitive detector (Figure 4(a)). Τhe accurate movement of the tip over the surface is achieved with piezoelectric elements in the *x*, *y*, *z* frame. The probe scans over the sample surface where any changes of the laser spot position on the detector are recorded and acted upon by feedback electronics, resulting in the accurate representation of the sample surface (Figure 4(a)).

One of the unique features of AFM is that once the user has found a region of interest or interesting feature, the cantilever tip can be used to apply a user-determined force on the sample. In AFM force spectroscopy, with careful calibration of cantilever spring constant, applied forces can be used to compress the sample, generating a force vs. indentation data. By using the AFM nanoindentation procedure [13–15], the stiffness/elasticity of the specimen can be recorded and Young's modulus maps of the sample's surface can be generated [16–20]. Additionally, further research is ongoing for the improvement of the existing mathematical models that are used for the acquisition of quantitative data from AFM modes [21].

## 2. Atomic Force Microscopy: Imaging Modes

Many other microscopes have different kinds of “modes” in order to extract more information about a surface. For example, SEM utilize secondary or backscattered electrons in order to image and provide information on topography and chemical composition, respectively. Optical microscopes can operate in a brightfield, darkfield, polarized, or phase contrast mode, depending on the optics that are used. Similarly, the AFM can operate in a number of different modes (Figures 4(b)–4(g)).

In the *tapping mode* (also known as intermittent mode, dynamic contact mode, and AC mode), the cantilever is tuned to its resonant frequency at a measured amplitude (Figure 4(b)); any changes in the oscillation amplitude are recorded as the tip scans the surface. Imaging with this dynamic mode is considered very gentle on sample surfaces, capable of high resolution, and almost all lateral forces are eliminated [3, 22].

In the *contact mode*, the tip is always in contact with the sample surface at a user-determined constant force (Figure 4(c)). Here, the user can track rougher, stiffer surfaces better and faster than the tapping mode. *Friction mode* imaging (Figure 4(d)) is a form of static mode (contact mode). This mode is appropriate for measuring the friction of a surface as the side to side twisting of the cantilever by a torque, measured as the probe raster scans along the surface. In the frictional force mode, here, the torsional changes (frictional features) in the photodetector are recorded simultaneously with the vertical changes (topographic features). Most AFM users will use this to gain qualitative frictional contrast of their surfaces; however, with appropriate calibration of the lateral cantilever spring constant [23], a surface frictional coefficient can be calculated.

During the tapping mode, simultaneously with topography images, phase images can be acquired [7]. In *phase imaging*, the system monitors the phase lag between the signal that drives the cantilever oscillation and its output signal (Figure 4(e)). Phase images can be used for assessing variations in composition, adhesion, and viscoelastic properties of surfaces [20, 24].

Other tapping mode-based characterizations include the *Kelvin probe* (Figure 4(f)) and *magnetic mode* (Figure 4(g)). Kelvin force microscopy (KFM) is one among a number of electrical characterizations that AFM can carry out. In this technique, a contact potential difference is measured between a conductive AFM probe and the sample of interest. This contact potential difference is negated by the application of a direct external bias of the same magnitude; which is then equal to the difference in work function between the tip and sample [25]. Magnetic imaging mode can image magnetic domain structures of a surface when a magnetic-coated cantilever scans the surface [26]. Here, the oscillating probe first scans the surface to get topographical features, but then, the probe elevates off the surface by a small distance and the attraction/repulsion is recorded.

AFM can also operate in a *noncontact mode* where the AFM tip does not touch at all the sample surface [27], but this mode is not frequently used for biological sample characterization.

In the *mechanical mapping mode*, the AFM measures the sample stiffness, in terms of Young's modulus values, through the nanoindentation technique. In AFM nanoindentation, the AFM collects indentation-force curves on the sample of interest while these curves are fitted using linear-elastic contact mechanical models, such as the Hertz model, in order to estimate Young's modulus [7]. It should be noted that the selection of the contact mechanics models to transform force-indentation curves into elastic moduli is the most fundamental and longstanding issue [28]. Modulus maps can then be formed by indenting in an organized array over a surface [16]. This technique can be very slow, as for example, a 64 × 64-pixel modulus map, with one indentation per second would take over an hour. Most AFM manufacturers have developed fast force mapping modes to speed up this procedure where indents are taken at kHz magnitude, meaning a 512 × 512 mechanical map can be taken in minutes. However, at this level of loading rate, the modulus results lack accuracy when indenting viscoelastic time-dependent materials. Further, the tip radius, which is vitally important for the modulus calculation, will be increasing during the imaging procedure.

To avoid this tip blunting effect during nanoindentation mapping, new mechanical imaging techniques have emerged. *Contact resonance* is a very useful mechanical mapping technique for materials ranging between 50 MPa and 200 GPa and consists of oscillating the samples, while undertaking a contact mode image. To date, this technique has been used only in few biological materials (for instance, for bone material [29]) although this technique does work well in air or under aqueous conditions [30].

In the *multifrequency*, *AFM* employs the detection of multiple cantilever frequencies (higher harmonics and/or higher flexural eigenmodes) which provides information concerning the tip-sample nonlinearity [31, 32]. Multiharmonic mode uses alterations in the amplitude, the phase of the oscillator, and other relevant harmonics in order to provide quantitative local property maps [30, 33]. Multiharmonic, like bimodal AFM which requires the simultaneous excitation of the first two eigenmodes of the cantilever, enables the simultaneous mapping of Young's modulus and the deformation and the topography of the sample [34]. As a result, these modes can be used for investigating complex cellular and biomolecular systems, in health and disease, and offering a detailed quantitative multiparametric characterization [35].

*Viscoelastic mapping* is another very novel mode that has its roots in research on multifrequency and bimodal AFM. This technique is a dynamic force-based mode that provides both imaging of the topography and maps of nanomechanical properties of soft-matter surfaces [36, 37]. Here, the cantilever is oscillated at two eigenmode frequencies—the first mode records the surface features and loss tangent data (amplitude modulation AM), while the second mode records frequency variations (frequency modulation FM) which relates to stiffness. AM-FM offers nanomechanical information of the specimen such as Young's modulus and contact stiffness [38] and has several advantages including fast scanning, high spatial resolution, and low forces applied to the sample. Although these modes are quite new, they have been used for investigating several biological samples, including proteins; small biological fibrils, like amyloid fibrils; lipid membranes; and viruses [39–42]. Also, it has been demonstrated that malign and benign cell lines present significant differences in their viscoelastic response [43].

## 3. Combined Imaging and Coupled AFM Systems and Their Applications

### 3.1. General

Although AFM presents high sensitivity and resolution on imaging and investigating biological samples at the nanoscale, it can lack other important information such as cellular components and biochemical functions [4, 44].

For example, optical microscopic imaging, especially using fluorescence, is another high-performance research tool that can provide complementary information. Fluorescence imaging can reveal the localization and quantify intracellular molecules and functions even at the level of a single molecule. Using specific fluorescence labelling, images of molecular mechanisms of biological functions can be acquired with high temporal resolution. Also, due to fluorescence intrinsic sensitivity to local microenvironment, valuable information on molecular specificities of cell structure can be obtained. However, as fluorescence imaging spatial resolution is limited by diffraction, its combination with AFM can produce images and provide us with information with high spatiotemporal resolution and biochemical specificity.

Generally, the combination of AFM with other microscopic imaging modalities can produce high-quality scientific information that cannot be achieved by using just one microscope.

### 3.2. AFM and Optical Microscopic Imaging

Optical microscopic techniques are often used in order to gain access to complementary information to AFM studies [45–47]. Using microscopies that utilize light in order to acquire images, such as simple optical, fluorescent, and laser scanning confocal microscope (CLSM), valuable information can be obtained concerning cellular process and interactions. Fluorescence microscopy incorporates fluorescent probes that either are accumulated in cellular components or are highly sensitive to environmental changes such as alterations of calcium ions and hydrogen ions. Because of its intrinsic sensitivity, fluorescence microscopy can image even a single fluorescent molecule if the background is not fluorescent [48].

CLSM uses a pinhole approach to spatially limit out-of-focus light or glare from reaching the image detector. In that way, only the image of the focal plane is acquired providing better spatial resolution not only in *x*-*y* plane but also across the *z*-axis [49, 50]. Furthermore, by incorporating machine vision techniques and with the appropriate calibration, not only qualitative but also quantitative data are obtained [51].

**Figure 5:**
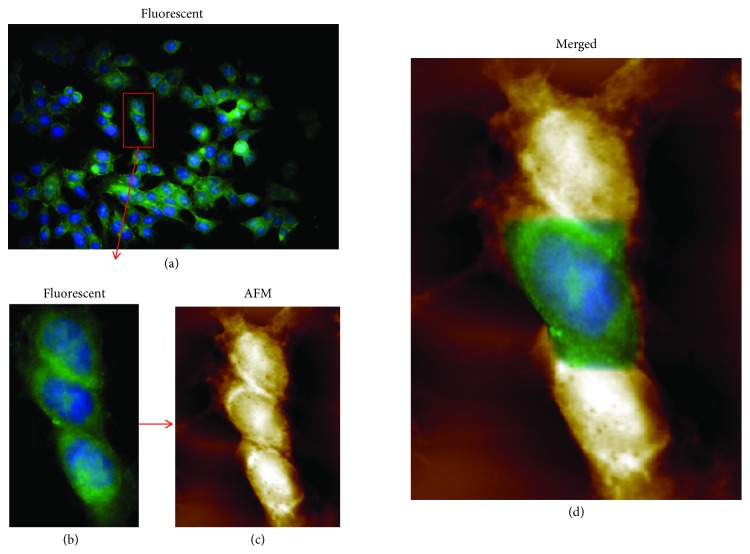
Combination of AFM with fluorescent microscopy: (a) MCF-7 breast cancer cells imaged with fluorescent microscopy (the blue color corresponds to DAPI staining that stains the cell nucleus and the green color represents antivasodilator-stimulated phosphoprotein (VASP) antibody. VASP is an actin-polymerization regulator which interacts with cell-ECM adhesion protein Migfilin and regulates cell migration [237]), (b) fluorescent image of three MCF-7 cells, (c) AFM image of the same cells, and (d) merged fluorescent and AFM images (unpublished data obtained by Andreas Stylianou in Cancer Biophysics Laboratory with an Olympus BX53 fluorescent microscope equipment, an Olympus XM10 monochrome CCD camera (1.4 megapixels), an UPlanFL N microscope objective lenses (40x/0.75 and 100x/1.30 oil), and a Molecular Imaging-Agilent PicoPlus AFM system (now known as 5500 AFM, Keysight Technologies)).

In recent years, an increase in the number of studies that combine AFM with optical microscopic techniques has been published. The combination of the two modalities can provide unique information from a single cell (Figure 5) to a tissue level. Staunton et al. have developed a novel technique that combines AFM with a confocal microscope in order to explore the mechanical properties of soft heterogeneous three-dimensional samples [52]. They used metastatic breast adenocarcinoma cells invading into collagen hydrogels, determining the elastic properties of both the cell and the extracellular matrix. Cells that are actively invading or fully embedded into the collagen matrix were found to be stiffer than cells lying on the matrix surface. This stiffening is Rho-associated protein kinase (ROCK) dependent, indicating that actomyosin contractility is a key player in the first phase of metastasis. On softer gels, enhanced invasion was observed either due to matrix softening by invading cells as a result of active rearrangement or proteolysis of adjacent fibers or because intrinsically softer tissues are preferable for invasion.

Stylianou et al. combined information from AFM with an optical and fluorescent microscope to study the tumor microenvironment such as fibroblasts (FBs) and cancer-associated fibroblasts (CAFs) [53]. In pancreatic tumors, treatment is reported to be blocked by desmoplasia, a cancer-specific type of fibrosis. Desmoplasia is a result of the interaction between stromal cells and tumor microenvironment. According to their results, CAFs express myofibroblast characteristics, such as *α*-smooth muscle actin expression, cell elongation, and lamellipodia formation, and are softer than FBs. Transforming growth factor beta (TGF-*β*) increases cell stiffness (Young's modulus) of both types of fibroblasts and elongation, cell spreading, lamellipodia formation, and spheroid invasion only in CAFs. In another study, Cazaux et al. have used AFM to mechanically stimulate, in a controlled manner over time and space, single immune cells and fluorescence imaging to follow the consequences of the contact through calcium fluorescent probes [54]. But they have also used light through a photoactivatable small G protein, Rac protein, to stimulate single T cell while recording cellular mechanical response. Canale et al. presented a review article trying to reveal the toxic interplay between lipid membranes and pathological protein aggregation in neurodegenerative disorders such as Alzheimer's and Parkinson's disease based on AFM studies [55]. In the study of Wegmann et al., neuroblastoma cells (N2a) and scrapie-infected mouse neuroblastoma (ScN2a) cells were studied with both AFM and immunofluorescence microscopy [56]. AFM imaging revealed a smooth surface topography of normal cells while scrapie-infected cells presented fibrillar aggregates on their surface. Immunofluorescence imaging, using a cellular prion protein (PrP) antibody (anti-PrP), revealed that PrP is homogeneously distributed in normal cells while it tended to segregate in preferential domains in ScN2a cells. Combining surface and fluorescence information, the authors concluded that the aggregated signal in SCNa2 cells is due to the structural changes at the cell surface. Finally, Kuyukina et al. using a hybrid imaging microscope, consisted of AFM and CLSM, studied bacterial viability at a single-cell level [57]. Fluorescent microscopy revealed that bacteria had compromised cell membranes when they were exposed to different organic solvent stresses. Combined information showed that changes in size, surface/volume ratio, and roughness were possible resistance mechanisms.

### 3.3. AFM and Second Harmonic Generation (SHG) Imaging

Except for fluorescence imaging, very promising microscopic modalities for biological imaging are based on nonlinear optics. These microscopic techniques can provide high-resolution, three-dimensional images without the use of fluorescent labelling. In that way, structural and dynamic images of living cells are acquired in a noninvasive manner. Among them, second harmonic generation (SHG) has been used in combination with AFM for investigation of biological samples. SHG occurs when two photons of the frequency *ω* interact with a nonlinear medium and are converted into a new photon with twice the energy (2*ω*). As a nonlinear process, only noncentrosymmetric structures such as collagen are able to emit SHG signals [58]. Stylianou et al. proposed the combined AFM and SHG information as a valuable tool to understand the nonlinear optical properties of collagen and in the long-term SHG to be used as a nondestructive imaging modality to monitor collagen-related diseases [59]. They have developed thin collagen films with controlled characteristics that can help in modelling the collagen matrix in a variety of pathological conditions such as thermal injury or mimic the structure of the collagen matrix in different tissues and conditions. AFM was used for thin film characterization while the SHG signal was correlated and interpreted with the predetermined characteristics of the films. Navab et al. investigated integrin *α*11*β*1, a stromal cell-specific receptor for fibrillar collagens that is overexpressed in CAFs, as a regulator of cancer stromal stiffness and promoter of tumorigenesis and metastasis in non-small-cell lung cancer [60]. Images acquired with SHG signals provided information on the quantification of collagen organization while AFM provided measurements of Young's modulus of the cancer-associated stroma. Their findings showed that the *α*11 signaling pathway in CAFs is closely related with collagen cross-linking, with stiffness and with organization of fibrillar collagen matrices. In another study, Robinson et al. performed a quantitative analysis of 3D extracellular matrix (ECM) remodelling that is involved in a large number of physiopathological processes in biology [61]. Pancreatic stellate cell interactions with ECM were studied in a 3D environment via ECM remodelling. AFM studies provided collagen topography, mechanical properties, and stiffness, while SHG microscopy in combination with image analysis techniques was used to observe and acquire images of collagen structure and topology and to further quantify collagen fiber changes in terms of alignment and thickness. Their results demonstrated that the increased ECM stiffness observed in fibrosis can be correlated with ECM remodelling. Also, Chevalier et al. worked with enteric neural crest cells (ENCCs) *in vitro* and during colonization of the gastrointestinal tract in chicken and mouse embryos [62]. They used AFM to study the influence of the stiffness of the environment on ENCCs both *in vitro* and *in vivo* and SHG to study its structure. The AFM results revealed that although initially the mesenchyme of the gut was soft, its stiffness was gradually increased during ENCC colonization. Complementary to these findings, SHG showed a gradual organization and enrichment of collagen fibers in the developing gut that are associated with the abovementioned stiffness. Furthermore, Liu et al. suggested the use of a cell-based system to generate a mechanoresponsive cell system (MRCS) that responds specifically to mechanoenvironmental cues to sense and kill cancer metastasis [63]. SHG imaging reported high collagen expression colocalized with cancer metastasis. Collagen networks were observed to be more linearized in cancer regions than in noncancer ones. AFM revealed an increased stiffness in cancer regions that are heterogeneous. Combination of AFM and SHG findings suggest a strong correlation between collagen cross-linking and increased tissue stiffness at the metastatic sites indicating the presence of a distinctive cancer mechanoenvironment.

### 3.4. AFM and Scanning Electron Microscopy (SEM) Imaging

AFM and SEM are two of the most commonly used nonoptical scanning microscopes that can provide us with surface imaging and characterization with high resolution. SEM has an excellent depth of field while AFM has poor depth of field, but excellent contrast on flat samples. SEM is able to measure the chemical composition of surface features, and AFM can measure its physical properties. If only image acquisition time is taken into account, SEM is faster than AFM but it produces 2D images while AFM can provide us directly with sample height information.

In most of the correlative microscopy research, more detailed information can be obtained by exploiting the advantages of both SEM and AFM [64–66]. For example, Mahmoud et al. performed a comparative study between AFM and SEM on healthy human liver parenchyma [65]. Hepatic architecture was mapped using both imaging modalities. AFM visualized the cellular structure, nucleus, and chromatin granules whereas SEM revealed other structures not observed by AFM. Pavlova et al. used both AFM and SEM to highlight their diagnostic and screening capabilities in kidney cancer based on pathomorphological changes in patient's erythrocytes [66]. Kaul-Ghanekar et al. on another combined study investigated the cellular morphology along with surface roughness [67]. They explored the effect of the tumor suppressor protein SMAR1 on cell lines and on tumor sections of mouse and human breast cancer of different grades. SMAR1 was found out to be a significant modulator of surface roughness and cytoskeletal volume. Finally, they suggested that SMAR1 might be used as a phenotypic differentiation marker between healthy and cancer cells. Also, Iyer et al. found, using combined AFM and SEM, different brush layers on the surface of normal and cancerous human cervical epithelial cells [68]. These brush layers are important for interacting with the environment and can be taken into account in characterization of cancer cells by means of forces and mechanical parameters. In another study, Volakis et al. used a mouse xenograft model to study the effect of myoferlin, a protein involved in membrane dynamics, on tumor formation and local invasion [69]. SEM along with image analysis quantified cell surface area, as well as the number and length of lamellipodia and filopodia. AFM was used to image and determine Young's modulus, a measure of cell stiffness that was found to be reduced in myoferlin-deficient cells and the integrity of the cytoskeletal network.

## 4. AFM and Collagen

### 4.1. General

As it was mentioned in the previous sections, AFM provides the ability to acquire topographic images at the nanoscale combined to the mechanical property determination at specific regions. Hence, the topographical features of surfaces can be simultaneously acquired with its material stiffness. Novel AFM techniques, like bimodal AFM, have increased the nanoscale characterization capabilities of AFM to detect compositional variations in soft specimens, like proteins and cells [34]. Due to its high resolution, AFM has been widely used for the characterization of biomolecules such as fibrous and globular proteins. The main role of fibrous proteins is to provide mechanical stability [70]. Typical examples of fibrous proteins are the collagen and the amyloid fibrils, which are discussed in the next sections of this review.

AFM has been also extensively used for the mechanical characterization of globular proteins (i.e., proteins with a spherical shape and surface irregularities). Globular proteins present structural complexity [70], and its mechanical testing is a challenging procedure. Specifically, the classic models of applied mechanics, such as the Hertz model, provide several limitations in the analysis; thus, they can only be used approximately. Typical examples of globular proteins studied using AFM are the lysozyme, the bovine carbonic anhydrase II, and the lactate oxidase. In particular, Radmacher et al. investigated the viscoelastic properties of single lysozyme molecules (absorbed on mica). For the theoretical analysis of the results, they used the Hertz model and Young's modulus of lysozyme was determined to be equal to 0.5 GPa [71]. In addition, for the investigation of the mechanical properties of bovine carbonic anhydrase II (BCA II), the modification of the Hertz model (which was proposed by Tatara [72]) was used. In the absence of Guanidine-HCl, Young's modulus of the native protein was determined to be equal to 75 MPa [73]. The mechanical properties of lactate oxidase (which has applications in bioanalytical devices for lactate determination) were determined by analyzing the fitted data obtained from the force curves (provided by AFM) to the Hertz model for conical and spherical indenter geometries. Young's modulus values in the case of lactate oxidase were calculated in the range 0.5-0.8 GPa [74].

### 4.2. Collagen

Collagens are the most abundant proteins in mammals and comprise almost the 30% of total cell protein in mammalian cells [75]. The collagen superfamily of vertebrate consists of more than 50 collagen and collagen-like proteins [76, 77]. The different members of the collagen superfamily have different shapes and sizes but the hallmark of a collagen is its monomer tropocollagen, which is composed of 3 polypeptide chains that form a right-handed triple-helical structure [76]. Each of these polypeptide chains contains a region with the repeating amino acid motif (Gly-X-Y), where X and Y can be any amino acid—12% of tripeptides have sequence Gly-Pro-Hyp; charged residues like arginine, lysine, and aspartic/glutamic acid make up approximately 15-20% of all residues in the monomer [78, 79].

**Figure 6:**
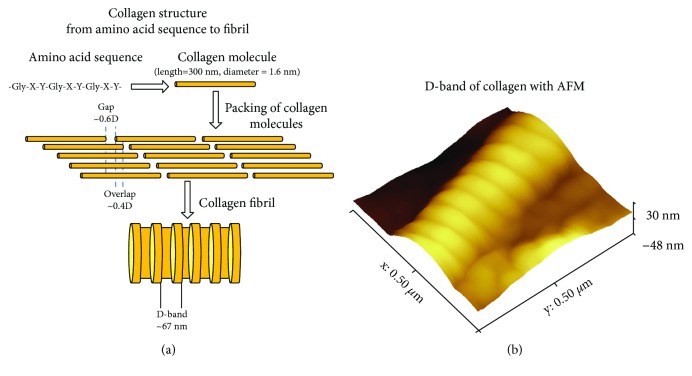
Collagen structure: (a) the collagen structure from the amino acid sequence to fibril. In this panel, the amino acid sequence, the collagen molecule, the packing of collagen molecules, and the structure of a collagen fibril are presented. (b) A collagen fiber D-band periodicity from a collagen thin film imaged using atomic force microscope (CPII Veeco-Bruker Microscope) in the tapping mode (adapted from [90]).

The human body contains about 28 different collagens, among which fibrillar collagens and especially collagen type I are of most interest [76, 80]. Collagen type I is the major protein in the extracellular matrix [75], and it is characterized by unique properties such as self-assembly, biocompatibility, biodegradability, and nontoxicity [81]. Collagen type I molecules form rod-shaped triple helices assembled to form fibrils [77, 82], which are then properly aligned in order to form bundles and fibers [77, 82] (Figure 6(a)). Interestingly, the molecules are packed in a quarter-staggered fashion so as to form the D-band (also known as D-periodicity), which is a repeating banding pattern of about 67 nm (Figure 6(b)), depending on the tissue [75, 76, 83–86]. The fibrils of collagen type I are the elementary building blocks in many collagen-rich tissues [87, 88], while it presents different morphological functions (including tissue mechanical strength and scaffolding to cell migration) in different tissues [75, 77, 89]. Furthermore, collagen has been identified as a unique biomaterial for the formation of novel bioengineering interventions [75, 90–92]. AFM characterization (imaging and mechanical property measurements) has been extensively performed in pure collagen or collagen rich-tissues as AFM does not destroy the fibrillary structure of collagen and can offer information from collagen molecules to separated fibrils/fibers [81, 93]. AFM has been used for investigating a number of different issues concerning collagen, from collagen structure to collagen-related pathological conditions and collagen optical radiation or cell interactions.

The structural and the mechanical properties of collagen at the nanoscale under various conditions have been extensively studied using AFM [5, 22, 90, 94]. The major advantage of AFM in collagen investigation comparing to other techniques is its ability to provide information regarding nanotopographical features such as the D-band periodicity (the collagen fibril consists of an alternating gap and overlapping regions with a highly reproducible D-band periodicity of approximately 67 nm) [17]. Simultaneously, AFM can provide data regarding the mechanical response of selected regions at the nanoscale. Hence, AFM is a suitable tool for the detection of local structural and mechanical variations of a single collagen fibril, under several external factors [11, 95]. The unique ability to combine information regarding nanotopographical and nanomechanical features using AFM in real time has led to groundbreaking solutions for the investigation of collagen [84, 96].

Thus, AFM has been used in several investigation areas related to tissues which contain collagen and has been proven a valuable tool in the field of histology and cytology [97]. Furthermore, AFM has been used for the investigation of collagen fibril structural variations at the nanoscale which are related to various pathological issues such as diabetes [98]. In addition, AFM has been increasingly used in the investigation regarding the influence of radiations arising either from nature or from medical activities (e.g., UV irradiation, radiofrequencies) on tissues which contain collagen [99, 100]. For example, the influence of UV irradiation on collagen at the nanoscale is a crucial issue regarding the general populations' health, due the chronic exposure of human skin in sunlight. As a result, the information provided at the nanoscale using AFM techniques has opened new insights into collagen research and the correlation of collagen nanofeature alterations with pathological situations.

#### 4.2.1. Collagen-Ultraviolet (UV) Radiation Interactions

The clarification of the interactions of skin with sunlight is of crucial importance since it is an intrinsic constituent of human life [101]. Solar radiation is made up of infrared, visible, and ultraviolet (UV-C, UV-B, and UV-A) radiation and exposure of the human body (i.e., skin, eyes) to sun and especially to UV can be harmful and lead to sunburn, photoaging, corneal damage, and carcinogenesis [102, 103]. Additionally, UV irradiation is applied for material science purposes, including for sterilizing and cross-linking biomaterials [104–106]. As UV can induce a number of alterations in collagen properties, including structure, chemical stability, and mechanical properties [107–111], AFM techniques have been extensively used for investigating UV-collagen interactions.

It has been shown that the irradiation of pure collagen with UV-C decreases the surface roughness [112–114], which is a crucial parameter in cell-surface crosstalk [115, 116] as modification in roughness alters the surface that is available for cell adhesion and growth [117]. The decrease of surface roughness due to UV irradiation has been demonstrated not only in pure collagen samples but also in collagen-based blends, including collagen-poly-vinyl alcohol, collagen-poly-vinyl pyrrolidone, and collagen-poly(e-caprolactone) [112, 118, 119]. Although surface roughness was modified through UV-C irradiation, it has been shown that for the same dose, collagen fibrils retained their characteristic D-band structure [114]. The D-band periodicity is important for the fibril mechanical properties and the cell-collagen crosstalk, while it has been correlated with specific pathological conditions [84, 86, 120, 121]. Furthermore, AFM nanoindentation was used in order to investigate the effects of UV irradiation on the gap and overlapping zones of collagen D-band [17]. It was found that UV irradiation has a significant effect on the height level differences between the overlapping and gap zones, while the fibril stiffness (in terms of Young's modulus) in the D-band zone was reduced, which was associated with the UV-induced polypeptide chain scission. Moreover, it has been demonstrated that cells' behavior is affected when cells are cultured on UV-irradiated collagen-based substrates [114, 122, 123]. It has been shown that cells can normally spread on collagen-based substrates that were UV-irradiated with doses relevant to those that are used for sterilizing scope cells, but when the irradiation time was increased, the cells were characterized by abnormal growth (in terms or cell shape) [114]. In addition, it has been demonstrated that in some cases, UV irradiation of collagen supported cell growth and enhanced cell viability [111, 122, 123]. As AFM studies did not reveal significant modifications in topography when collagen is irradiated with low UV doses, the alterations in cell behavior have been suggested to be a consequence of either mechanical property alterations and/or chain scission [100, 124].

#### 4.2.2. Low-Level Laser (LLL) Therapy Collagen Interactions

Low-level laser therapy (LLLT), or photobiomodulation [125], applies low-level (also referred and as low-power) lasers for the treatment of several pathological conditions or abnormalities, including fibromyalgia, osteoarthritis, tuberculosis, temporomandibular joint disorders, and wound healing that presents increased interest. In the case of wound healing, it has been suggested that the underling mechanism, which remains partially unknown, includes the increase of mitochondrial function, ATP, RNA, and protein synthesis [126]. Subsequently, these lead to increased oxygen consumption and enhanced synthesis of the reduced form of nicotinamide adenine dinucleotide (NADH) and ATP. As a result, a promotion of the cellular metabolism was observed along with enhanced wound healing mainly through the increase proliferation of fibroblasts and their ability to produce collagen. Although the collagen role in the progress has been identified, the LLL-collagen interactions remain partially understood. Stylianou and Yova in 2015 used AFM techniques in order to investigate the effects that LLL (irradiation in the red region, 661 nm) has on collagen and the influence on cell culturing [127]. After LLLT irradiation, no statistical significant alterations were found in the nanoscale topographical characteristics of the collagen substrates (collagen thin films), but a negative effect (abnormal cell shape) was found on the fibroblasts that were cultured on the irradiated collagen substrates. As the modulation of fibroblast shape and behavior was recorded in the absence of measurable surface alterations, it was suggested that these effects are not influenced by the surface properties of the substrate through the surface contact guidance mechanism [128, 129]. These results offer new insights toward the clarification of the LLLT mechanism and the role of collagen in this procedure.

#### 4.2.3. Nanomechanical Properties of Collagen

**Figure 7:**
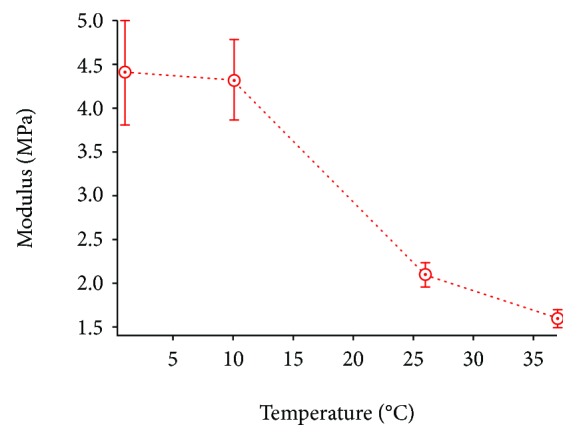
Moduli of a single individual collagen fibril at varying temperatures in PBS buffer (Colin Grant; unpublished data).

Extended studies focused on collagen using AFM techniques have been performed during the last decades due to the fact that many parts of the mammalian body contain collagen in the form of fibers (e.g., the skin, bones, cartilage, and tendons contain collagen fibers). Typical measurements of collagen fibers have been performed by Minary-Jolandan and Yu [11]. In their research, the mechanical differences on collagen fibrils due to the D-band periodicity were revealed (Young's modulus values were calculated to be ~1.2 GPa for the gap and ~2.2 GPa for the overlapping regions). The mechanical heterogeneity due to the D-periodicity was also confirmed by Kontomaris et al. [17]. Mechanical differences in the periodic banding of collagen from a rat tail tendon was also reported by Grant et al. [84]. However, Grant et al. took both a static and dynamic indentation approach to show that the topography had variances not only in elastic moduli but also in the time-dependent behavior at physiologically relevant frequencies (0.1-2 Hz). In addition, Strasser et al. determined Young's modulus of collagen fibril type I in the range 1.2 ± 1 GPa [130] and Yadavalli et al. in the range 1.03 ± 0.31 GPa [131]. In the same order of magnitude were the results presented in the research conducted by Heim et al. (i.e., Young's modulus values varied in the range 1-2 GPa) [132]. On the contrary, the results provided by Wenger et al. differ greatly comparing to the previous values (5-11.5 GPa) [133]. As mentioned, the key advantage of AFM is to carry out imaging and characterization under aqueous conditions, so it was shown that the nanomechancial properties of bovine Achilles tendon collagen fibrils changed by three orders of magnitude (1.9 ± 0.5 GPa to 1.2 ± 0.1 MPa) when going from ambient to buffer conditions [134]. Further, it was shown that reconstituted bovine Achilles tendon collagen can be mechanically tuned by altering the imaging buffer [78]. It was also shown that in imaging the same fibrils in air and under buffer, the diameter doubled in size. Then, the mechanical properties of collagen approximately doubled with addition of 1 M monovalent chloride salts, further increased by reducing the pH, and increased by a factor of 100 by testing under 100% ethanol [78]. Most AFM studies test the compressive mechanical properties by nanoindentation; however, it is possible to carry out tensile testing on collagen fibrils [135]. Here, it was also shown that human collagen fibrils have reduced sensitivity to saline and pH compared to reconstituted collagen fibrils. As well as varying the aqueous conditions, the effect of temperature has also been carried out—nanomechanical properties of the same individual collagen fibril varied from 4.5 MPa at 1°C to 1.6 MPa at 37°C (Figure 7). This demonstrates the importance of carrying out nanomechanical property characterization of a biological matter under the most relevant physiological condition.

However, the determination of mechanical properties at the nanoscale is a challenging procedure. It is obvious from the results presented above that the extended range of values on Young's modulus values, even on the same sample, has been recorded (e.g., see the wide range of Young's modulus values regarding collagen in Table 1). The basic reasons for the wide range of Young's modulus values (even when the same sample is tested) are as follows:

- The restrictions which are provided by the classic models of applied mechanics, commonly used for the analysis of the data obtained using AFM
- The uncertainties in the experimental determination of critical magnitudes such as the spring's constant determination and the exact shape and dimensions of the AFM tip
- Different used protocols for the sample preparation (e.g., testing in air or in liquid environment)

An analytical discussion of the approximations regarding the AFM indentation technique on biological samples is presented in “Approximations of the AFM Nanoindentation regarding Biological Samples and Biomaterials at the Nanoscale” in Supplementary Materials (available here).

#### 4.2.4. Collagen in Tissue Samples

As previously discussed, collagen is the most abundant protein in the human body and is found in most tissues that requires some form of biomechanical structure/strength. AFM analysis can be carried out to both image not only the collagenous structure of the tissue but also the nanomechanical properties. The sclera (white of the eye) has been investigated to show that the mechanical properties vary considerably on removing the episcleral layer to reveal the collagenous stromal layer [136]. The skin has the remarkable regenerative capacity to be able to heal itself in a complex cascade of biochemical and cellular remodeling processes. It was recently shown that the nanomechanical and viscoelastic properties of the upper dermis from the human skin change following wound healing [96]. Imaging revealed a variance in the orientation of collagen fibrils in scar tissue compared with health skin tissue, but also the scar tissue is stiffer than healthy tissue and also healthy tissue retains a higher dissipative characterization [96]. Similar dynamic/static nanoindentation was also carried out on porcine blood vessels [137]. The outer layer of the blood vessels (tunica adventitia) is a collagen layer that helps prevent overexpansion. Here, the adventitial layer of the porcine aorta vs. pulmonary arteries was compared, under aqueous conditions at 37°C. It was demonstrated that the aorta exhibited larger viscoelastic and dissipative characteristics, which makes sense as the aorta is under higher pressure compared with the pulmonary artery [137].

## 5. AFM and Specific Pathological Conditions

### 5.1. Articular Cartilage and Osteoarthritis

#### 5.1.1. Cartilage

Articular cartilage is a connective tissue, which provides wear-resistant joint motion and low friction. It covers the ends of long bones and its thickness is in the range 1–3 mm. The main function of articular cartilage is to absorb and distribute the applied loads due to the joint movements. In addition, it provides protection to subchondral bone from damage. The functionality of articular cartilage is engendered by its collagen fiber meshwork and extrafibrillar proteoglycan-rich matrix which is highly hydrated [138]. Loss of collagens or proteoglycans or structural changes may lead to a water content change and is a result to several damages to the tissue. As it has been previously reported, a loss of proteoglycans from the hydrated gel which is followed by an irreversible collagenolytic degradation of the fibrils results in damages regarding the collagen meshwork and as a result to the development of osteoarthritic cartilage [139].

Various methods are able to provide direct *in vitro* observation of cartilage under physiological conditions. Typical examples are the optical microscopy [140, 141], visual inspection, and histology [142, 143]. However, these methods are limited to a spatial resolution of approximately 200 nm. In addition, in the case of electron microscopy, ultrastructural details of articular cartilage at a molecular level can be provided, but the procedure requires dehydration and chemical fixation of the cartilage combined with metal staining or sputtering. Thus, the cartilage specimen cannot be examined at near physiological conditions. Moreover, optical and electron microscopy cannot provide measurements of the mechanical properties of articular cartilage.

Τhe most direct method for stiffness measurements is the classic compression testing. However, the compression test requires sample manipulation (e.g., the sample's surfaces have to be highly parallel and the cartilage has to be cut off the bone). On the other hand, macroindentation procedure does not require sample manipulation; hence, typical indentation testing devices can be used to evaluate the quality of the health or the disease state of articular cartilage [144–146]. These devices usually employ indenters with diameters in the range 1-2 mm in order to evaluate the correspondence of the sample to a specific applied load. However, stiffness measurements at the millimeter scale are not sensitive to detect pathological conditions (e.g., early-stage osteoarthritis) or changes due to aging [144, 146, 147].

#### 5.1.2. Osteoarthritis

As it was already mentioned, various methods have been used for the determination of cartilage's pathological conditions; however, they are limited to specific conditions (e.g., measurements at the microscale or measurements at nonphysiological conditions) or they provide only specific information (e.g., imaging or mechanical properties). On the contrary, AFM provides the possibility of simultaneous imaging and mechanical property measurements (i.e., Young's modulus maps) on a micro- or nanometer scale in specimens that are near physiological conditions. Articular cartilage presents an interesting behavior depending on the level that is being tested. This interesting behavior has been revealed using AFM. In particular, at the micrometer scale, it behaves as a uniform and non-structured material. Hence, at the microscale, it presents an overall stiffness value which can be used as an initial approximation in order to evaluate its properties. In addition, it must be noted that cartilage can be modeled as a poroviscoelastic material; hence, the overall stiffness measurements provide an aggregate modulus, both viscous and elastic. As it has been previously reported, the aggregate modulus ranges between the values 1 MPa and 60 MPa depending on the loading conditions (~1 MPa for low-frequency conditions (<0.1 Hz) and ~60 MPa for high-frequency loading (~40 Hz)).

Moreover, Young's modulus at specific regions of articular cartilage at the microscale or at the nanoscale can be calculated using AFM. As it has been previously reported, the elastic modulus of the articular cartilage intercellular matrix exhibits a depth-dependent increase. Specifically, Young's modulus value at the superficial zone is approximately 0.52 MPa and at the calcifying deep zone ~1.69 MPa [148, 149]. The major advantage of AFM comparing to traditional methods is that is able to provide information regarding the functionality of articular cartilage. Thus, using AFM, diseases such as osteoarthritis can be detected at the initial stages. AFM has been used by many researchers as a tool for the diagnosis of cartilage's pathological conditions at early stages [14, 15, 150]. In particular, Stolz et al. [15] calculated Young's modulus of normal articular cartilage at the microscale (2.6 MPa) and nanoscale (0.021 MPa). The results of Loparic et al. are in the same order of magnitude. The microstiffness of articular cartilage was determined to be 1.3 ± 0.4 MPa and the nanostiffness 22.3 ± 1.5 kPa for cartilage's the proteoglycan gel (PG gel) and 184 ± 50 kPa for the collagen meshwork [150]. Stolz et al. investigated osteoarthritis at early stages and proved that microstiffness of articular cartilage does not present differences comparing to healthy cartilage. Hence, osteoarthritis at the early stages can be only detected at the nanoscale. In particular, at the nanoscale, nanostiffness was calculated to be 83 kPa in the case of normal cartilage and 5.6 kPa in the case of grade 3 osteoarthritis [14]. Hence, these findings proved the ability to detect early stages of osteoarthritis using AFM and have opened a new prospect for the use of AFM as a clinical tool. In particular, Stolz et al. tested AFM in real clinical activities; articular cartilage biopsies from seven patients who suffer from osteoarthritis (the age of the patients was in the range 62-96 years) were examined [14]. The result of this research has revealed an increasing softening of articular cartilage at the nanoscale with progressing osteoarthritis at a given age. It must be noted that all the detected changes at the initial stages of osteoarthritis could only be detected at the nanoscale.

In addition, Stolz et al. have shown that biomechanical and morphological changes occurring in articular cartilage during normal aging differ comparing to the ones occurring during osteoarthritis progression. The cartilage softening during osteoarthritis progression is due to the disintegration of collagen meshwork [14]. The stiffening of the collagen meshwork at the nanoscale can only be detected using AFM [14]. Hence, the ability to detect changes at early stages of osteoarthritis and distinguish these changes from the ones that occurred due to normal aging has opened an exciting prospect of using AFM as a tool in real clinical activities.

### 5.2. Cancer

Cancer progression has been associated with alterations in cancer cells and tumor microenviroment (TME) components [151, 152]. Alterations in cancer cells that make them differ from normal cells include changes in cell morphology, reproduction, communication, adhesion, cell-to-cell or cell-to-ECM interactions, cell invasion/metastasis, and even cell death. Recently, it has been demonstrated that cancer cells have different nanomechanical properties than normal cells and alterations in the mechanical properties of cells play a crucial role during malignant transformation [152, 153]. TME consists of the tumor blood and lymphatic vessels, the stromal cells (FBs and CAFs), the ECM (including mainly collagen and hyaluronic acid), and a number of other soluble factors. Tumors have the tendency to modify their microenvironment in a way that promotes tumor growth and progression. For instance, in desmoplastic tumors, like pancreatic and breast cancers, the interactions between TME components lead to desmoplasia, which is characterized by the presence of CAFs and the overproduction of ECM components [154, 157]. This desmoplastic reaction leads to TME stiffening which increases the compressive mechanical forces in the tumor interior [113, 155, 156]. Furthermore, the crosstalk between TME and cancer cells influences a number of cancer cells' properties, including cell proliferation, migration, and cell invasion through surrounding tissues. Consequently, the understanding of cancer cells and TME alterations during cancer progressions is of utmost importance, in order to develop novel and more effective anticancer therapies or diagnostic techniques. There are several studies correlating the influence of the mechanical properties of the ECM and tumor progression [157, 158]. For instance, it has been demonstrated that caveolin-1 (Cav1, the major component of endocytic caveola plasma membrane, promotes among others the force-dependent contraction and TME stiffening [158]. AFM arises as a key tool in this research area, providing many novel research results [4, 159, 160]. AFM can be used as a unique technique in order to study cancer cell properties (such as growth, invasion, and metastasis), TME alterations, and cancer tissue progression. Generally, its ability to work on live cells with high resolution and to assess nanomechanical properties (e.g., Young's modulus properties) has made AFM a valuable technique in the field of cancer research. Apart from *in vitro* experiments of AFM on cancer cells, AFM has been used for studying the nanomechanical properties of TME components [4] and cancer tissue biopsies with great potentials [160–162]. In this section, we first present the use of AFM for characterizing the nanomechanical properties of cancer cells and its use for discrimination between healthy and cancer cells. Then, we discuss the use of AFM for investigating the nanomechanical properties from tissue biopsies and the possible application for early cancer diagnosis.

#### 5.2.1. Cancer Cells

*(1) High-Resolution Imaging*. Although it has been demonstrated that cancer cells have different characteristics and properties than normal cells, they remain poorly identified or understood [163]. AFM techniques have already been used for studying cancer and normal cells in terms of morphology, cells' surface, pericellular activity, proteolytic activity, exosomes, cell-cell or cell-ECM interactions, and nanomechanical properties.

One of the key characteristics of AFM and one of its major advantages are its ability of high-resolution imaging. Although other techniques, like optical and fluorescence microscopy, can offer significant information concerning cancer cell morphology at the single-cell level, AFM can provide information at the nanoscale concerning cell morphology and surface. For instance, AFM has been used for studying invadopodia which are cellular processes extended by the cells and play a significant role in cancer cell invasion and metastasis. Chasiotis et al. and Fillmore et al. used AFM to image the surface of human glioblastoma brain tumor cells (T98) on collagen substrates during invasion process [164, 165]. They demonstrated that invadopodia present an unusual nanomorphology and their results offered new insights concerning cell-substrate interactions. Additionally, AFM high-resolution imaging has been used for the characterization of single exosomes [166], which play a significant role in tumor proliferation and tumor microenvironment modulation [167–169]. Sharma et al. applied AFM techniques in order to investigate the exosomes from normal and cancer cells and showed that cancer cell-derived exosomes possess surface nanofilaments that may help in cell-cell communication [170]. Also, AFM can be applied to investigate specific cell activities that seem to be crucial for cell metastasis, as pericellular proteolytic activity which is important for TME remodeling and cancer cell invasion [171]. AFM can be used for assessing differences in average height, volume, and molecular weight distribution of pericellular matrix proteins in TME [171–173].

In the research area of cell-substrate interactions, AFM can provide unique information concerning the interactions of normal and cancer cells with culture substrates and obtain new data about cell-ECM interactions. It has been demonstrated that nonmetastatic breast cells adhered less to mineralized ECM secreted by osteoblasts than metastatic cells [174]. Furthermore, AFM has been used for investigating the mechanical response of cancer cells on different substrates. Park et al. studied prostate and breast cancer cells that were cultured on nanoscafolds and showed that different cancer cell lines have distinct response to substrate characteristics such as size and nanomechanical properties [175]. Additionally, AFM can be used for studying cell-cell interaction. For instance, Puech et al. studied melanoma cell line during the binding process on human umbilical vein endothelial cells [176], while Laurent et al. investigated the adhesion strength between endothelial cells and cancer cells and demonstrated that the more invasive cells formed the strongest bonds with endothelial cells [177]. Hoffmann et al. used AFM in order to study the interaction forces among tumor cells and natural killer cells and demonstrated that in order to separate natural killer cells from cancer cells, the required forces are higher when the killer cell receptor 2B4 is activated [178].

*(2) Mechanical Properties of Cancer Cells*. Another area that presents a significant research interest is the use of AFM for assessing cancer cell nanomechanical properties. It can provide extremely high precision on the force applied to the cell, live and fixed ones, under natural conditions (e.g., in liquid conditions with temperature control), and it can provide quantitative mechanical measurements simultaneously to high-resolution imaging. As alteration in cytoarchitecture is associated with cancer progression [164, 179, 180] and the cells' mechanical properties play a crucial role in cell motility, metastasis, and growth [181, 182], the investigation of the nanomechanical properties of cancer cells, in comparison with normal ones, is of particular scientific interest.

One of the first demonstrations of the AFM mechanical measurements on cancer cells was performed in 1993 by Weisenhorn et al. on lung cancer cells, but with a quite big variation in their Young modulus values. Afterwards, force-volume mode was used in order to provide enough statistics on embryonic cancer cells by Goldmann et al. [183, 184]. Although the study of single cancer cells can offer significant information concerning their mechanical properties, in 1999, the pioneer work of Lekka et al. demonstrated that the comparative investigation of cancer and normal cells can provide unique data [185, 186]. Bladder cells with different levels of malignancy were used, and the results demonstrated that cancer cells were softer than normal cells. This was the first study that demonstrated that AFM can be used for distinguishing cancer cells from normal cells in terms of cell deformability. In order to further correlate the cell malignancy with their deformability, a number of studies have been performed on different cell lines, including breast [161, 187], prostate [161, 188], and oral [189] tumor cells, showing that cancer cells are softer than normal cells in terms of Young's modulus values. Furthermore, Cross et al. investigated cells that were obtained from pleural effusion of patients and showed that metastatic cells are softer than benign cells in clinical samples [190], while in a number of studies, it has been demonstrated that cells derived from normal or nonmalignant tissues present a similar pattern [153]. All these studies suggest that the stiffness of tumor cells (in terms of Young's modulus) can be considered as a diagnostic method for distinguishing them from normal cells [161]. Additionally, recently, it was demonstrated that CAFs were also softer than normal FBs and present increased invasive properties [53]. Furthermore, it has been demonstrated than malign and benign cell lines present different viscoelastic properties [43]. More specifically, cancer cells are characterized a substantially larger loss tangent than benign cells [43]. On the other hand, it has been suggested that cell softening is a cell-type-specific response [191] and that this cell characteristic alone fails to serve as a universal indicator for metastatic progression [192]. Consequently, more research is demanded toward this direction so as AFM to be used as a diagnostic tool for detecting cancer cells and distinguish them from normal ones in real clinical practice.

#### 5.2.2. Cancer Tissue

Although in the previous section we present that cancer cells are softer than normal cells, it is a common belief that tumors are stiffer than their host tissue [162]. Tumor stiffening is caused by the increase in ECM composition, mainly collagen type I and collagen crosslinking during cancer progression [193–195], and it is the major biomechanical property of solid tumors that clinicians and patients can feel during palpation. Concerning the mechanical properties of tumor, AFM seems to be a valuable tool for assessing its nanomechanical properties with high spatial resolution. Although it has been presented that AFM has the potentials to be a unique cancer diagnostic tool [162, 180, 196], the work toward this direction is limited [160, 197–199].

**Figure 8:**
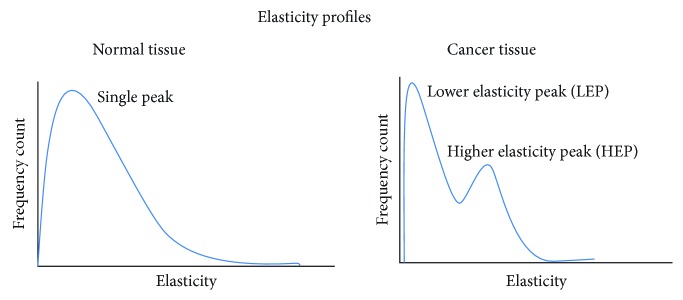
Schematic representations of predicted AFM elasticity measurements in normal and cancer tissues.

One of the first studies on this area was performed on snap-frozen mammary tissues, and the results demonstrated that the malignant epithelium was stiffer than in isolated breast cancer cells and that the ECM adjacent to the epithelium progressively stiffens [198]. In 2012, Lekka et al. investigated tissue samples from patients with endometrioid carcinoma of the uterine, breast, and vulvar cancers and their results demonstrated that these tissues were softer than the nonneoplastic regions [161]. In a pioneer work, in the same year, Plodinec et al. demonstrated that AFM can be used for assessing unique nanomechanical fingerprints from human breast biopsies that can be used for cancer breast diagnosis [162]. Their results revealed that normal and benign tissues present a uniform stiffness (in terms of Young's modulus) distribution, while malignant tissue was characterized by two distinguishable maxima. The same maxima were reported by Tian et al. in 2015 in human liver tissue and were referred as lower elasticity peak (LEP) and higher elasticity peak (HEP) (Figure 8) [199]. Also, in this study, the different stiffness distributions and maxima of other tissues (including esophageal cancer, clear cell renal cell carcinoma, colon cancer, and papillary renal cell carcinoma) were reported.

In 2016, Ciasca et al. demonstrated that AFM can assess nanomechanical fingerprints of human glioblastoma and meningothelial meningioma and AFM techniques can be applied for tumor brain grading [200]. In the same year, Ansardamavandi et al. developed clustering algorithms in order to divide and categorize the derived stiffness (in terms of Young's modulus values) measurements [197]. With their technique, they achieve to divide the measurements into three categories (the cellular, the noncellular, and the fibrous part of the tissue) and demonstrated that AFM can be combined with computational methods for identifying more than two different components of the tissue. In 2017, Cui et al. used AFM for investigating the nanomechanical fingerprints of cervical cancer and cervical intraepithelial neoplasia [201]. The research results demonstrated that both healthy and cancer samples present bimodal distribution of Young's modulus values with similar values of LEP, but cancer HEP values demonstrated that cancer tissues were significantly stiffer than the healthy controls. Consequently, HEP values could be used as a nanomechanical biomarker for cancer diagnosis. Also, in 2017, Minelli et al. applied an operator-independent neural network in order to identify the nanomechanical fingerprints of brain cancer [202]. Their technique achieves to identify and distinguish cancer from healthy tissue in a fully automated fashion.

The results so far have demonstrated that AFM is arising a novel technique for assessing the nanomechanical properties of cancer tissue and has the potential to provide a novel tool for early cancer diagnosis.

### 5.3. Alzheimer's Disease

#### 5.3.1. Amyloid Fibrils

The study of protein fibrils is an important subject in various research fields and disciplines. A very interesting fibrous protein complex is amyloid fibrils which are highly ordered fibrillar structures assembled from either peptides or unfolded proteins [203]. The deposition of amyloid, in the form of amyloid plaques, has been associated with a number of degenerative diseases, while the amyloid structure has also been found in many functional proteins that are not related with a specific disease. As the involved mechanisms remain unknown, the clarification of the mechanisms of fibrillation, the structural features of the amyloid fibrils, and their physical and nanomechanical properties will help to reveal their biological role [203]. AFM and its capabilities for nanomechanical characterization of biological samples arise as a very significant tool for the study of the amyloid fibrils. For instance, Roeters et al. used AFM in combination with other techniques (such as X-ray powder diffraction and IR spectroscopy) for studying the aggregation of the intrinsically disordered protein alpha-synuclein (*α*S) into amyloid fibrils [204]. These amyloid fibrils have been correlated with the pathology of Parkinson's disease. Their results demonstrated that the structure of *α*S fibrils varies as a function of ionic strength, and they argued that this sensitivity to the ionic strength might form the basis of differences in *α*S-related pathologies. Also, in the last years, many researchers have focused on the self-assembly of amyloid peptides and proteins at interfaces since it will provide significant information for understanding the mechanism of some neurodegenerative diseases [205]. The AFM has been employed in this area and so far, the results demonstrate that interfaces play an important role in peptide assembly [205]. Furthermore, very recently, Watanabe-Nakayama and Ono used high-speed AFM (HS-AFM), which allows the video imaging of the conformational changes of individual molecules, in order to investigate the structural dynamics of individual amyloidogenic protein assemblies [206].

#### 5.3.2. Alzheimer's Disease

As it was already mentioned, the formation of fibrous particles is associated with a number of specific diseases. One of these, which have a great social impact, is Alzheimer's disease (AD). Patients with this disease presents neuritic plaques and vascular deposits consisting from fibrous aggregates of the protein amyloid-*β* (A*β*). More specifically, the hydrophilic molecules of A*β* accumulated outside of the neural cells and result in the formation of these highly toxicity amyloid plaques [207]. These amyloid plaques are composed of amyloid fibrils and small oligomers, which are insoluble protein aggregates [208]. Although the aggregation of the A*β* protein has been correlated with neurotoxicity, the involved mechanisms remain unclear [7]. Soon after the development of the AFM, it was demonstrated that the AFM is a powerful tool for studying the A*β* amyloids and the AFM can provide unique information for understanding the structural origins of this complex neurodegenerative disease [7]. For instance, in 1996, AFM was used to investigate the self-assembly and the surface structure of the resultant fibrils [209, 210]. AFM has been applied in many studies since then for studying Α*β* amyloids with several research purposes. Some of the more recent research results use advanced, sophisticated, and coupled AFM techniques. Furthermore, AFM has been used for studying *β*-amyloid structure in liquid conditions [211] and *β*-amyloid aggregation as a function of concentration [212]. Also, Connelly et al. investigated the ion channel mechanism and pore structures of the amyloids of AD pathology by using AFM [213], while several studies study the relationship between the formation of *β*-amyloid fibrils and toxicity in AD [214]. Furthermore, Song et al. used AFM in combination with fluorescence spectroscopy so as to study the interactions between vanillin and A*β* polypeptide [215]. Their results demonstrated the depolymerization of A*β*1-42 aggregates by vanillin in a dose-dependent manner, and the authors suggested that vanillin may be a potential pharmacological agent for the treatment of AD. In another study, Han et al. developed a sophisticated AFM-based technique to evaluate the amyloid precursor protein (APP) cleavage mechanism at the nanomolecular level, as the clarification of the APP cleavage mechanism is crucial for the development of new AD therapeutic agents [216]. Also, Banerjee et al. studied isolated cross-linked A*β*42 trimers (A*β* oligomers) with HS-AFM which enabled the visualization of the structural dynamics of the oligomers at nanometer resolution on a millisecond time scale. According to the researchers, the nanoscale characterization of the A*β* oligomer structure and dynamics can lead to the development of novel oligomer-specific therapeutic agents. In other recent works, Li et al. used real-time AFM combined with molecular dynamics simulations as a novel approach to investigate the amyloid nanostructures formed by a potential pentapeptide inhibitor [217], while Han et al. applied AFM in order to probe the interactions between A*β* and two kinds of antibodies [207].

### 5.4. Viruses and HIV

#### 5.4.1. Viruses

AFM has opened a new prospect regarding the investigation of the physical properties of viruses [218, 219]. The research regarding the structural properties of viruses and virus mechanics facilitates the engineering of their physical properties in order to improve their applications in bionanotechnology and molecular biology [220]. In particular, AFM provides the ability to acquire topographic images of biological objects at the nanometer scale (such as the viruses). The first imaging attempt regarding viruses, using scanning probe microscopy (STM), was provided by Baró et al. [221]. The virus particle that was visualized was the bacteriophage Φ29. But as in STM imaging the sample has to be electrical conductive, the particle was covered with a metallic layer that is far from its physiological conditions. This limitation has been overcome using AFM, which does not require electrical conductivity. AFM has been widely used for the topographic property determination of viruses, partially disassembled viruses, viral capsids, nucleic acids, etc. [222–225]. In addition, AFM can be used in order to investigate the viral infection in live cell process [226–229] and for the determination of the interaction forces between viruses and other molecules [220].

Viruses are solid-state objects; thus, the determination of their mechanical properties is possible. For almost all “empty” capsids, Young's modulus has been determined under the assumption that the capsid is homogeneous and hollow with idealized geometry [220]. In addition, the shell thickness and the empty capsid's size are considered to be similar to the real capsid's size [218, 220]. For Young's modulus determination, the thin elasticity theory and finite element analysis on the model particle are usually used. However, the validity of this approach for Young's modulus determination is under discussion [230]. A different approach for the determination of the mechanical properties of viruses is based on the consideration of the virus particle and the AFM cantilever as two ideal springs in series. In this case, the “spring constant” of the particle *k*_*p*_ can be determined using the following equation: *k*_ef_^−1^ = *k*_*p*_^−1^ + *k*_*c*_^−1^ where, *k*_ef_ is the value of the effective spring's constant (which is calculated from the slope of the force vs. distance curves) and *k*_*c*_ is the cantilever's spring constant. However, despite the fact that stiffness, as it is calculated using the spring constant, is an object's property, it does not depend only on the material but also depends on the object's dimensions and geometry.

Moreover, an alternative method for the mechanical property determination of viruses is the structural strength determination. In the most of the cases, the linearity of the force vs. distance curves is lost when a specific force is applied. The loss of the linearity is due to the mechanical failure or buckling in the particle. In addition, it must be noted that for the mechanical property determination of particles, a number of load-indentation curves are used. The force experiments usually do not cause permanent damages to the particles. However, a big number of indentation experiments may result in a nonlinear mechanical response or in mechanical failure. This behavior is caused by the material fatigue of the particle.

#### 5.4.2. HIV

A typical example of a virus in medicine, with great significance and social impact that can be tested using the AFM methods, is the human immunodeficiency virus (HIV). HIV is an enveloped retrovirus with a genetic material that in the first phases of development is in a single-stranded RNA form [231]. In the mature phase of development, the viral genomic RNA is encapsidated within a cone-shaped capsid [231]. HIV capsid is a conical shell with a length ~100-120 nm which is formed during viral maturation by the assembly of about 1500 capsid protein molecules (these molecules are organized into 250 hexamers and 12 pentamers approximately). The presence of these pentamers induces the curvature necessary to form the cone shape of the capsid [232–234].

The calculation of the stiffness of the capsid is mandatory for the assessment of any mechanical model regarding the capsid's uncoating. Several experiments for the determination of the mechanical properties of HIV 1 particles using AFM have been performed during the last decade [220, 231, 235, 236]. In particular, Ramalho et al. applied the nanoindentation technique to measure the stiffness of empty capsids independently from the viral envelope. In particular, in vitro assemblies of wild-type and mutant recombinant HIV-1 capsid protein and isolated and mutant HIV-1 cores (i.e., filled capsids) were tested. This research resulted that the mutant assemblies were significantly stiffer than the wild-type assemblies. Pang et al. investigated the mechanism under which the physical properties of viral particles change during maturation and the effects of these changes in the viral lifestyle [235]. Their results indicated that HIV presents a severe reduction in particle stiffness during maturation that is mediated by the viral envelope protein. Kol et al. compared the mechanical properties of mature and immature HIV viruses using AFM nanoindentation [236]. The spring constant of the mature HIV-1 virus was calculated equal to 0.22 N/m while the spring constant of immature HIV-1 virus was calculated equal to 3.15 N/m. In addition, Young's moduli were found to be 0.44 GPa and 0.93 GPa for the mature and the immature HIV-1, respectively, which means that the immature HIV-1 virus is stiffer than the mature one. The research regarding HIV is of great importance due to the fact that the mechanical softening as a result of maturation of HIV-1 (which is due to the loss of Env-Gag interactions) is probably a necessary condition before the infection occurrence [220].

## 6. Conclusions

AFM is a unique tool for nanocharacterization, including high-resolution imaging and nanomechanical measurements, of biological samples under different environmental conditions. New advances of AFM enable the simultaneous imaging with other modalities offering new perspectives in the field. A plethora of studies so far have demonstrated the abilities of AFM for assessing unique nanocharacteristics of biological samples that can be correlated with different pathological conditions. Consequently, AFM stands out as the ideal research instrument for exploring the detection of pathological conditions even at very early stages, making it very attractive in the area of bio- and nanomedicine.

## Figures and Tables

**Table 1:** Typical examples of Young's modulus values of collagen in the literature.

References	Young's modulus on collagen		
	Collagen source	Contact model used	Young's modulus values (GPa)
[17]	Type I collagen from bovine Achilles tendon	Oliver & Pharr model	0.74 - 1.43
[131]	Type I atelocollagen from calf skin	Hertz model	1.03 ± 0.31
[11]	Type I collagen from bovine Achilles tendon	Hertzian model, modified for cylindrical sample	1.2 - 2.2
[133]	Type I collagen fibrils from rat tail tendons	Oliver & Pharr model	3.75 - 11.5
[130]	Type I collagen fibrils from calf skin	Hertz model	1.2 ± 1

## Abbreviations

3D: Three dimensional
AD: Alzheimer's disease
AFM: Atomic force microscopy
APP: Amyloid precursor protein
ATP: Adenosine triphosphate
CAFs: Cancer-associated fibroblasts
CLSM: Laser scanning confocal microscope
ECM: Extracellular matrix
ENCCs: Enteric neural crest cells
FBs: Fibroblasts
HIV: Human immunodeficiency virus
HS-AFM: High-speed AFM
ILK: Integrin-linked kinase
KFM: Kelvin force microscopy
LLLT: Low-level laser therapy
MRCS: Mechanoresponsive cell system
NADH: Reduced form of nicotinamide adenine dinucleotide
PrP: Prion protein
SEM: Scanning electron microscopy
SHG: Second harmonic generation
SPM: Scanning probe microscopy
STM: Scanning tunnelling microscopy
TEM: Transmission electron microscopy
TME: Tumor microenvironment
UV: Ultraviolet
VASP: Vasodilator-stimulated phosphoprotein.

## Supplementary Materials

Supplementary Materials Approximations of the AFM nanoindentation regarding biological samples and biomaterials at the nanoscale.
