# Supplementary material for: Atomic Force Microscopy on Biological Materials Related to Pathological Conditions
Source: Scanning. 2019 May 12;2019:8452851. doi: 10.1155/2019/8452851 (PMC6535871; doi:10.1155/2019/8452851)
Supplement: Supplementary Materials — Approximations of the AFM nanoindentation regarding biological samples and biomaterials at the nanoscale. [file 8452851.f1.pdf]

Supplumentary

Approximations of the AFM nanoindentation regarding biological samples and biomaterials at the nanoscale

As it was previously stated the determination of the mechanical properties at the nanoscale using AFM is a challenging procedure due to the various uncertainties provided by the theoretical analysis and experimental errors. Many researchers during the last decades used AFM to determine the mechanical properties of biosamples at the nanoscale. However, the results provided on the same biological sample (e.g. the same type of collagen fibrils or the same cell type) by different laboratories presented large variation (Schillers and others, 2017). The reasons for the presented variations are the differences in data processing protocols and the instrumental errors (Schillers and others, 2017).

In particular, the classic models of applied mechanics can only approximately be used for the mechanical characterization of biological samples and biomaterials at the nanoscale (typical experiments on biological samples and biomaterials in which classic models of applied mechanics, regarding the data processing, have been used are presented in **Supplementary Table 1**).

Supplementary Table 1: Applications in biomaterials and biological samples

Reference	Application	Applied model
Li et al., 2008	Mechanical properties characterization of Cancerous (MCF-7) and Binign (MCF-A) human breast epithelial cells	Hertz model
Efremov et al., 2014	The elastic properties of cells (with different level of cancer transformation) were investigated	Hertz model
Liang et al., 2004 (Liang and others, 2004)	Mechanical properties determination of unilamellar phosphatidylcholine liposomes.	Hertz model
Strasser et al., 2007	Experiments for the determination of the Young's modulus of collagen fibrils type I.	Hertz model
Janko et al., 2010	Structural and mechanical integrity investigation of collagen type I in mummified human skin.	Modification of the Hertz model (sphere – cylinder contact)
Wenger et al., 2007	Young's modulus determination of isolated collagen fibrils (type I) in air environment.	Oliver and Pharr model
Kontomaris et al., 2015a	The investigation of the effects of UV irradiation on collagen fibrils type I.	Oliver and Pharr model
Stolz et al., 2009	Young's modulus determination of porcine articular cartilage	Oliver and Pharr model
Stolz et al., 2004	Indentation experiments in mice and in patients' articular cartilage for the	Oliver and Pharr model

	determination of the Young's modulus in each case.	
Choi et al. , 2012 (Choi and others, 2012)	Biomechanical properties of human anterior lens capsules (ALCs) investigation for non-cataract and cataract groups using AFM.	Oliver and Pharr model
Darling , 2011	Indentation experiments in agarose gels, cells and articular cartilage for the Young's modulus determination.	Force scanning method (based on the linearized Hertz model).
Kontomaris et al., 2016	Investigation of the influence of low power red irradiation on collagen fibrils.	Force scanning method (based on the linearized Hertz model).

In particular, the most common model for the analysis of the data obtained using AFM is the Hertz model and its modifications. However, the Hertz model can be applied under the following assumptions (Fischer-Cripps, 2009; Kontomaris and Stylianou, 2017):

1. The sample is flat or it has spherical symmetry. In addition, the sample must be homogeneous and isotropic. However, the biological samples at the nanoscale present complex structure and topography and do not meet these requirements. Nevertheless, heterogeneous materials at the micro or at the nanoscale can be considered approximately as half spaces in the case that the AFM tip is small comparing to the sample's dimensions. In particular, Wenger et al. concluded that a collagen fibril can be approximately considered as a half space in the case that the fibril's radius is at least 5 times bigger than the tip radius (Wenger and others, 2007). However, as it was reported by Kontomaris et al. in this case (i.e. the fibril's radius is 5 times larger than the tip radius) the error in the Young's modulus calculation will be ~4.6% (Stylianos-Vasileios and others, 2018). An extended study which evaluates the errors arising by the consideration of a biological sample with cylindrical or spherical symmetry at the micro or at the nanoscale as a half space has been performed by Kontomaris et al. and correction factors have been provided in each case (Stylianos-Vasileios and others, 2018). Hence, in the case that the tip radius is comparable to the sample's radius, modifications in the Hertz model have to be made (e.g. sphere – cylinder contact) (Janko and others, 2010; Li and others, 2008). However, the half space approximation is reasonable in some cases. Typical examples of biological samples which can considered as half spaces are the benign (MCF – 10A) and cancerous (MCF – 7) human breast epithelial cells (Li and others, 2008) in the case that are being indented by tips with radius equal to several nanometers. On the contrary, in the case that a micro-sized spherical indenter is used the most appropriate choice is to consider these samples as spherical.
2. The tip-sample contact is frictionless and adhesionless. In the case that the contact presents significant adhesion, the JKR (Johnson–Kendall–Roberts), the DMT (Derjaguin–Muller–Toporov) and the Maugis models (Johnson, 1971; Johnson and Greenwood, 1997; Maugis, 1992) must be applied. In the case of large indenter radius, high adhesion forces and compliant samples the suitable model is the JKR. On the contrary, in the case of small indenter radius, small adhesion forces and stiff samples, the DMT model must be used. The

Maugis model applies in the transition regime between the JKR and DMT models.
3. The contact geometry has to be continuous, smooth and axisymmetric.
4. The indentation depth cannot exceed the 5-10% of the sample's thickness (according to the Buckle's rule (Persch and others, 1994)).

It is obvious that the mentioned assumptions can provide significant errors in the Young's modulus calculation. The approximations which are considered in order to apply the Hertz model and its extensions are less than ideal in the case of biomolecules (i.e. biomolecules are anisotropic with extended structural diversity and the biomolecule – tip contact is usually conforming and frictional (Kurland and others, 2012)). For this reason the Young's modulus values on the same kind of biomolecules (even in the cases of biomolecules that being tested in the same lab) present a wide range of values.

In addition, non-negligible errors arise from the experimental procedure. These errors are mostly caused by uncertainties of the exact shape and dimensions of the indenter, the approximate determination of the cantilever's spring constant and the deflection sensitivity determination (Schillers and others, 2017). In particular, the uncertainties provided by the ignorance of the exact AFM tip's dimensions and shape can provide errors in Young's modulus calculation approximately 20% (Kontomaris and others, 2015; Mateu, 2012). In addition, non – negligible are the errors provided by the spring's constant value. As it has been previously reported, using different methods for the spring's constant determination, can provide differences in the range 5-20% (Kontomaris and others, 2015; Mateu, 2012). Even when the thermal noise method is used (which is probably the most well known and accurate method) the error in the cantilever's spring constant can be 10-20% in the case of V-shaped cantilevers (Kontomaris and others, 2015).

In addition, it must be noted that non negligible are the errors provided by the deflection sensitivity value. As it has been previously stated the two main sources that provide errors in the Young's modulus calculation, in the case of biosamples, using AFM are the spring's constant determination and the determination of the deflection sensitivity (Wagner and others, 2011).

Also, it has been demonstrated that the AFM measurements of the elastic moduli in a force-indentation experiments of a thin soft layer is affected by the solid support of the specimen (Dimitriadis and others, 2002). This is particular important for the measurements of living cells attached to a solid support, such a petri dish or glass coverslips (Garcia and Garcia, 2018). According to Garcia and Garcia 2018the bottom effect elastic theory can be used to recover of the intrinsic mechanical properties of the cell (e.g. the Young's modulus) with independence of the stiffness of the solid support and they suggest that the use of sharp tips can reduce the bottom effect artifacts.

The capability to develop accurate protocols in order to obtain reproducible results between different laboratories will enable AFM as a powerful tool for clinical applications. For example it can be used as a powerful tool for the detection of diseased cells (Efremov and others, 2014; Lekka and others, 2012; Li and others, 2008; Rianna and Radmacher, 2016; Suresh and others, 2005; Trickey and others, 2000) or osteoarthritis at early stages (Stolz and others, 2009). For this reason efforts

are being made by many different laboratories across the world in order to obtain accurate and highly reproducible results in the case of biological samples. These efforts will convert AFM as a powerful clinical instrument for the determination of a wide of pathological conditions.

References

Choi S, Lee HJ, Cheong Y, Shin JH, Jin KH, Park HK, Park YG. 2012. AFM study for morphological characteristics and biomechanical properties of human cataract anterior lens capsules. Scanning 34(4):247-256.

Dimitriadis EK, Horkay F, Maresca J, Kachar B, Chadwick RS. 2002. Determination of elastic moduli of thin layers of soft material using the atomic force microscope. Biophysical Journal 82(5):2798-2810.

Efremov YM, Lomakina ME, Bagrov DV, Makhnovskiy PI, Alexandrova AY, Kirpichnikov MP, Shaitan KV. 2014. Mechanical properties of fibroblasts depend on level of cancer transformation. Biochimica et Biophysica Acta - Molecular Cell Research 1843(5):1013-1019.

Fischer-Cripps AC. 2009. The IBIS Handbook of Nanoindentation. Forestville NSW 2087 Australia: Fischer-Cripps Laboratories Pty Ltd.

Garcia PD, Garcia R. 2018. Determination of the Elastic Moduli of a Single Cell Cultured on a Rigid Support by Force Microscopy. Biophysical Journal 114(12):2923-2932.

Janko M, Zink A, Gigler AM, Heckl WM, Stark RW. 2010. Nanostructure and mechanics of mummified type I collagen from the 5300-year-old tyrolean Iceman. Proceedings of the Royal Society B: Biological Sciences 277(1692):2301-2309.

Johnson KK, K.; Roberts, A. . 1971. Surface energy and the contact of elastic solids. Proceedings of the Royal Society of London. A. Mathematical and Physical Sciences 324(1558):301-313.

Johnson KL, Greenwood JA. 1997. An adhesion map for the contact of elastic spheres. Journal of Colloid and Interface Science 192(2):326-333.

Kontomaris SV, Stylianou A. 2017. Atomic force microscopy for university students: Applications in biomaterials. European Journal of Physics 38(3):033003.

Kontomaris SV, Yova D, Stylianou A, Politopoulos K. 2015. The significance of the percentage differences of young's modulus in the AFM nanoindentation procedure. Micro and Nanosystems 7(2):86-97.

Kurland NE, Drira Z, Yadavalli VK. 2012. Measurement of nanomechanical properties of biomolecules using atomic force microscopy. Micron 43(2-3):116-128.

Lekka M, Pogoda K, Gostek J, Klymenko O, Prauzner-Bechcicki S, Wiltowska-Zuber J, Jaczewska J, Lekki J, Stachura Z. 2012. Cancer cell recognition - Mechanical phenotype. Micron 43(12):1259-1266.

Li QS, Lee GYH, Ong CN, Lim CT. 2008. AFM indentation study of breast cancer cells. Biochemical and Biophysical Research Communications 374(4):609-613.

Liang X, Mao G, Ng KYS. 2004. Probing small unilamellar EggPC vesicles on mica surface by atomic force microscopy. Colloids and Surfaces B: Biointerfaces 34(1):41-51.

Mateu MG. 2012. Mechanical properties of viruses analyzed by atomic force microscopy: A virological perspective. Virus research 168(1):1-22.

Maugis D. 1992. Adhesion of spheres: The JKR-DMT transition using a dugdale model. Journal of Colloid and Interface Science 150(1):243-269.

Persch G, Born C, Utesch B. 1994. Nano-hardness investigations of thin films by an atomic force microscope. Microelectronic Engineering 24(1-4):113-121.

Rianna C, Radmacher M. Cell mechanics as a marker for diseases: Biomedical applications of AFM; 2016.

Schillers H, Rianna C, Schäpe J, Luque T, Doschke H, Wälte M, Uriarte JJ, Campillo N, Michanetzis GPA, Bobrowska J, Dumitru A, Herruzo ET, Bovio S, Parot P, Galluzzi M, Podestà A, Puricelli L, Scheuring S, Missirlis Y, Garcia R, Odorico M, Teulon JM, Lafont F, Lekka M, Rico F, Rigato A, Pellequer JL, Oberleithner H, Navajas D, Radmacher M. 2017. Standardized Nanomechanical Atomic Force Microscopy Procedure (SNAP) for Measuring Soft and Biological Samples. Scientific Reports 7(1):5117

Stolz M, Gottardi R, Raiteri R, Miot S, Martin I, Imer R, Staufer U, Raducanu A, Düggelin M, Baschong W, Daniels AU, Friederich NF, Aszodi A, Aebi U. 2009. Early detection of aging cartilage and osteoarthritis in mice and patient samples using atomic force microscopy. Nature Nanotechnology 4(3):186-192.

Stylianos-Vasileios K, Andreas S, Anna M, Triantafyllos S. 2018. A discussion regarding the approximation of cylindrical and spherical shaped samples as half spaces in AFM nanoindentation experiments. Materials Research Express 5(8):085402.

Suresh S, Spatz J, Mills JP, Micoulet A, Dao M, Lim CT, Beil M, Seufferlein T. 2005. Connections between single-cell biomechanics and human disease states: Gastrointestinal cancer and malaria. Acta Biomaterialia 1(1):15-30.

Trickey WR, Lee GM, Guilak F. 2000. Viscoelastic properties of chondrocytes from normal and osteoarthritic human cartilage. Journal of Orthopaedic Research 18(6):891-898.

Wagner R, Moon R, Pratt J, Shaw G, Raman A. 2011. Uncertainty quantification in nanomechanical measurements using the atomic force microscope. Nanotechnology 22(45).

Wenger MPE, Bozec L, Horton MA, Mesquidaz P. 2007. Mechanical properties of collagen fibrils. Biophysical Journal 93(4):1255-1263.
